# The *14-3-3*σ gene promoter is methylated in both human melanocytes and melanoma

**DOI:** 10.1186/1471-2407-9-162

**Published:** 2009-05-27

**Authors:** Suhu Liu, Paul Howell, Suping Ren, Oystein Fodstad, Adam I Riker

**Affiliations:** 1 Dana-Farber Cancer Institute, Boston, MA, USA; 2 Mitchell Cancer Institute-University of South Alabama, Mobile, AL, USA; 3 Institute for Cancer Research, Norwegian Radium Hospital, Oslo University Hospital , Montebello, 0310 Oslo, and Faculty Division The Norwegian Radium Hospital, Faculty of Medicine, University in Oslo, Oslo, Norway; 4 Ochsner Cancer Institute, Department of Surgery 1514 Jefferson Highway, BH334 New Orleans, LA 70121, USA

## Abstract

**Background:**

Recent evidence demonstrates that 14-3-3σ acts as a tumor suppressor gene inactivated by methylation of its 5' CpG islands in epithelial tumor cells, while remaining un-methylated in normal human epithelia. The methylation analysis of 14-3-3σ has been largely overlooked in melanoma.

**Methods:**

The methylation status of 14-3-3σ CpG island in melanocytes and melanoma cells was analyzed by methylation-specific sequencing (MSS) and quantitative methylation-specific PCR (Q-MSP). 14-3-3σ mRNA and protein expression in cell lines was detected by real-time RT-PCR and western blot. Melanoma cells were also treated by 5-aza-2'-deoxycytidine (DAC), a demethylating agent, and/or histone deacetylase inhibitor, Trichostatin A (TSA), to evaluate their effects on 14-3-3σ gene expression.

**Results:**

14-3-3σ is hypermethylated in both human melanocytes and most melanoma cells in a lineage-specific manner, resulting in the silencing of 14-3-3σ gene expression and the active induction of 14-3-3σ mRNA and protein expression following treatment with DAC. We also observed a synergistic effect upon gene expression when DAC was combined with TSA. The promoter methylation status of 14-3-3σ was analyzed utilizing Q-MSP in 20 melanoma tissue samples and 10 cell lines derived from these samples, showing that the majority of melanoma samples maintain their hypermethylation status of the 14-3-3σ gene.

**Conclusion:**

14-3-3σ is hypermethylated in human melanoma in a cell-linage specific manner. Spontaneous demethylation and re-expression of 14-3-3σ is a rare event in melanoma, indicating 14-3-3σ might have a tentative role in the pathogenesis of melanoma.

## Background

14-3-3σ is a highly conserved acidic protein family, composed of seven isoforms in mammals. Of the seven known isoforms, 14-3-3σ appears to be the only one directly involved in human cancer [1]. The 14-3-3σ gene has been implicated in G2/M cell cycle arrest by p53 and acts as a tumor suppressor gene (TSG) in colorectal cancer [2]. 14-3-3σ (also know as Stratifin) was first identified as an epithelial cell antigen (HME-1) exclusively expressed in human epithelia. Recent evidence demonstrates that the 14-3-3σ gene promoter region is un-methylated in normal epithelial cells while inactivated via hypermethylation of its 5' CpG islands in epithelial malignancies. Gene silencing of 14-3-3σ by CpG hypermethylation has been found to occur in many human cancer histologies, including breast cancer [3], hepatocellular carcinoma [4], vulvar squamous neoplasia [5], gastric carcinoma [6], oral carcinoma [7], epithelial ovarian cancer [8], and prostate and endometrial carcinoma [9].

The methylation status of 14-3-3σ in melanoma has not been previously investigated. Thus, we wished to examine whether 14-3-3σ gene is aberrantly methylated in melanoma. We first examined the CpG island methylation status and gene expression of 14-3-3σ in normal human epidermal melanocytes (NHEM) and melanoma cells. We report that NHEM does not express significant levels of 14-3-3σ protein, nor is it expressed at the transcriptional level, primarily due to the dense hypermethylation of the 14-3-3σ gene CpG island. We show that the 14-3-3σ gene is methylated in most melanomas cells in a cell lineage-specific manner, with spontaneous demethylation and re-expression of 14-3-3σ a rare event in human melanoma.

## Methods

### Cell culture

The human melanoma cell lines, A375 (American Type Culture Collection, Manassas, VA), WM266-4 (Wistar Special Collection), Lox (established from a lymph node metastasis of a patient at the Norwegian Radium Hospital) [10] and C8161.9 (a highly metastatic, amelanotic melanoma cell line derived from an abdominal wall metastasis) [11] were grown in monolayer culture in RPMI 1640 with glutamine supplemented with 10% fetal bovine serum at 37°C in a humidified atmosphere consisting of 5% CO2 and 95% air. NHEM cell lines (Cambrex, Baltimore, MD) were cultured under the same incubator conditions, utilizing specialized melanocyte media (Cambrex) according to the supplier's recommendations. We established numerous melanoma cell lines from freshly excised clinical melanoma samples utilizing previously published techniques for tissue procurement and in vitro melanoma cell line growth and expansion [12]. These melanoma cell lines were further characterized by flow cytometry and/or cytospin preparation for cellular confirmation of melanoma cell purity of >99% (data not shown). All melanoma cell lines were derived from either thick primary or metastatic melanoma tissue samples.

### Tumor procurement

Over a 3-year period, we surgically procured tumor samples from patients with primary cutaneous melanoma (PM) and metastatic melanoma (MM). All samples were obtained under an Investigational Review Board (IRB) approved tissue procurement protocol (MCC#13448, IRB#101751; PSM# 990914-JM, 020318-JM). Upon surgical removal of the primary melanoma, a single surgical oncologist (A.I.R.) utilized a scalpel to macrodissect and procure a portion of the remaining primary tumor, with a similar technique utilized for grossly involved lymph nodes where the melanoma had completely replaced the lymph node. Samples were taken from non-necrotic areas of the tumor. The same process was performed for all distant metastases, careful to avoid surrounding tissues or stroma. All samples were cryopreserved in liquid nitrogen and stored within the Tissue Procurement Laboratory of the Moffitt Cancer Center, securely de-identified through a centralized database. We analyzed 4 thick PM's (>4 mm) and 16 MM samples composed of 12 bulky, macroscopic (replaced) lymph node metastases and 4 subcutaneous metastases.

### 5-aza-2'-deoxycytidine and Trichostatin A treatment of cells

Melanoma cells were seeded at a density of 5 × 10^5 ^cells/10 cm plate on day 1. Cell lines were treated with 5 μM DAC (Sigma) for 96 hours or 200 ng/mL TSA (Sigma) for 24 hours. For the combination treatment, cells were first treated with 5 μM DAC (Sigma) for 96 hours, then 200 ng/mL TSA were added and cells were treated for an additional 24 hours. Culture medium containing DAC or TSA was prepared fresh daily. At the end of treatment, the medium was removed and the RNA and protein were isolated for subsequent reverse transcription PCR and Western blot analysis.

### Nucleic acid isolation and bisulfite modification

Genomic DNA (gDNA) was isolated from tumor tissue and cultured cell lines, using Qiagen Blood and Cell Culture DNA Kit (Qiagen, Inc., Valencia, VA) and stored at -20°C before use. 0.5 μg gDNA was used for bisulfite treatment with the EZ DNA Methylation Kit (Zymo Research, Orange, CA) according to the supplier's protocol.

### Real-Time Quantitative Methylation-Specific PCR (Q-MSP)

Promoter methylation of 14-3-3σ gene was investigated utilizing quantitative, methylation-specific PCR (Q-MSP) [13]. Briefly, bisulfite-treated DNA was amplified using real-time PCR with oligonucleotide primers complementary to a region of the β-actin sequence that does not contain CpG dinucleotides but does contain non-CpG cytosines. From this, we were able to ascertain the amount of converted input DNA template for each sample. Hypermethylation of 14-3-3σ CpG islands was then examined with real-time PCR amplification of bisulfite-modified DNA using oligonucleotide primers as reported [3]. The performance of the Q-MSP primers was evaluated by running a standard curve and melting curve before they were applied for quantitative gene methylation analysis in tumor samples. All PCR reactions were carried out on an iQ5 real-time thermal cycler (Bio-Rad, Hercules, CA) at 95°C for 10 min followed by 40 cycles of 95°C for 15 s and 60°C for 60 s. Each PCR reaction was carried out in a 25 μl volume containing 2× SYBR Green PCR master mix (Applied Biosystems, Foster City, CA), 200 nM each primer and 1 μl bisulfite-modified gDNA. All reactions were carried out in duplicate. Bisulfite-converted, universally methylated human gDNA (Chemicon, Temecula, CA) and gDNA from MDA-MB-435 served as positive controls and bisulfite-converted gDNA from MDA-MB-231 and blank reactions with water served as negative controls as it has been shown that 14-3-3σ CpG island in MDA-MB-231 cell line was un-methylated [3].

### Methylation-specific sequencing (MSS) of 14-3-3σ CpG island

To further validate the MSP results, gDNA from NHEM and C8161.9 was bisulfite- modified and the CpG island in the first exon was amplified by PCR using the primers as reported [3] which produce a PCR product with 474 bp and containing 27 CpG dinucleotides. The PCR products were gel purified using QIAquick Gel Extraction Kit (Qiagen, Valencia, CA, USA), ligated into pCR2.1-TOPO vectors (Invitrogen) and transformed into DH5a-competent cells (Invitrogen). 10 colonies were chosen randomly. Plasmid DNA was purified and sequencing was carried out by MWG Biotech, Inc. (Huntsville, AL).

### RNA isolation and real-time reverse transcription PCR

Cultured normal melanocytes and melanoma cells lines were collected and dissolved in TRIzol^® ^(Invitrogen, Carlsbad, CA) and RNA was purified according to the manufacturer's recommendations. Purified RNA was then treated by DNase I (Roche, Indianapolis, IN) to remove genomic DNA contamination. First-strand cDNA synthesis was performed with 2 μg total RNA for each sample in a total volume of 20 μl using a high capacity cDNA archive kit (Applied Biosystems, Foster City, CA). The reverse transcription reaction was performed with random primers and incubated at 25°C for 10 min followed by 37°C for 120 min. Real-time PCR analysis of 14-3-3σ mRNA was performed using Assays-on-Demand Gene Expression Assays (Applied Biosystems, Foster City, CA) (assay ID: Hs00602835_s1) with GAPDH (assay ID Hs99999905_m1) serving as an internal control. All PCR reactions were performed in a total volume of 25 μl containing 2× TaqMan Universal PCR Master Mix (Applied Biosystems), 20× Assays-on-Demand Gene Expression Assay Mix and 40 ng cDNA. All assays were performed in triplicate and run on an iQ5 real-time thermal cycler (Bio-Rad, Hercules, CA) using the following conditions: 50°C for 2 min, 95°C for 10 min, and 40 cycles of 95°C for 15 s and 60°C for 1 min. Relative quantitation of the amplified products was based upon Ct values.

### Western blot analysis

Protein extracts were obtained from cell pellets utilizing standard procedures, fractionated on a 12% polyacrylamide gel, transferred to a nitrocellulose membrane and blocked for 1 h with 5% milk in Tris-buffered saline-T. 14-3-3σ was detected using anti-14-3-3σ monoclonal mouse antibody [1.N.6] (Abcam) in 5% milk in Tris-buffered saline-T incubated overnight at 4°C. The immunoreactive band (Mr 30 kDa) was visualized by the chemiluminescence kit (Immobilon Western; Millipore, Billerica, MA). Beta-Actin was used as a control.

## Results

### 14-3-3σ CpG island is hypermethylated in NHEM and most melanoma cell lines

**Figure 1:**
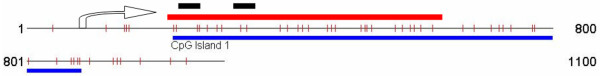
**CpG island promoter region of the 14-3-3 gene**. The CpG Island Searcher Program (Takai et al, 2003) identified a CpG island within the 14-3-3σ gene, downstream of the transcriptional start site. Arrow represents the transcriptional start site. Blue line depicts the CpG island. Red line indicates the area analyzed by methylation-specific sequencing and black lines represent the location of the methylation specific PCR primers.

**Figure 2:**
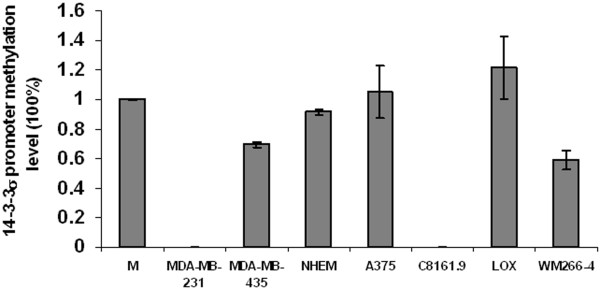
**Quantitative Methylation-Specific PCR analysis of 14-3-3σ gene methylation level in cell lines**. Error bars were defined as "mean +/- SD". M: universally methylated human genomic DNA as positive control.

**Figure 3:**
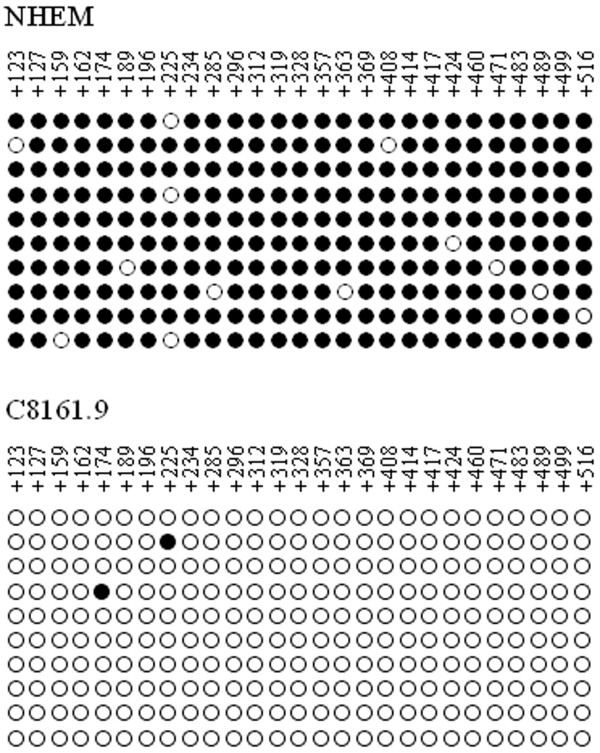
**Methylation-specific sequencing of 14-3-3σ DNA in cell lines NHEM and C8161.9**. Open circles represent un-methylated CpG dinucleotides; filled circles represent methylated CpG dinucleotides. Nucleotide positions are relative to transcriptional start site.

We first utilized Q-MSP to determine the methylation status of the 14-3-3σ gene in NHEM and several established melanoma cell lines, including A375, C8161.9, LOX and WM-266-4. Figure 1 shows a map of the CpG island for the 14-3-3σ gene (662 bp) as well as the PCR primers utilized for methylation analysis. As shown in Figure 2, consistent with previous reports [3], the 14-3-3σ CpG island was shown to be un-methylated in the cell line MDA-MB-231, but heavily methylated in MDA-MB-435. This result confirmed the accuracy of the Q-MSP method utilized here in evaluating 14-3-3σ CpG island methylation. Results from Q-MSP reveal that the 14-3-3σ CpG island was hypermethylated in NHEM as well as the melanoma cell lines A375, LOX and WM266-4, but un-methylated in C8161.9. To further validate the Q-MSP results, methylation-specific sequencing (MSS) was performed upon genomic DNA derived from NHEM and C8161.9 cells. Utilizing MSS, we evaluated the 27 CpG dinucleotides contained within the CpG island of the first exon from the 14-3-3σ gene. Figure 3 shows that the MSS results are correlative with the MSP results, demonstrating that all 27 CpG sites within the 14-3-3σ gene are heavily methylated in NHEM but un-methylated in C8161.9.

### Correlation of methylation status with 14-3-3σ gene expression

**Figure 4:**
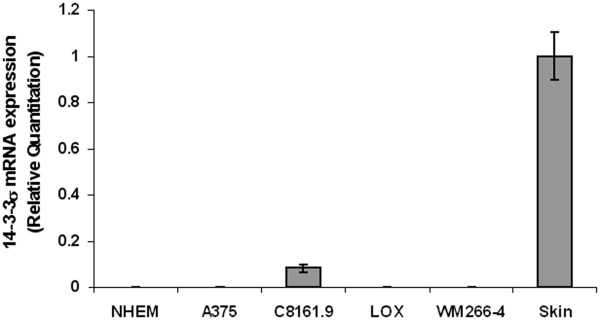
**Real-time PCR analysis of 14-3-3σ mRNA expression**. Error bars were defined as "mean +/- SD".

**Figure 5:**
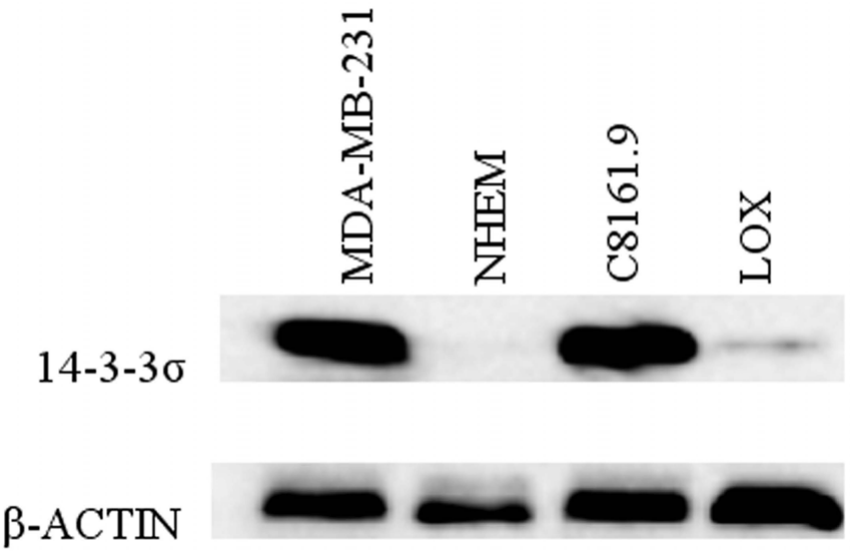
**Western blot detection of 14-3-3σ protein in cell lines**. Protein from MDA-MB-231 was utilized as positive control.

Expression of 14-3-3σ mRNA and protein were evaluated with real-time RT-PCR and Western Blot analysis, respectively. As shown in Figure 4, 14-3-3σ mRNA expression is significant in C8161.9 but undetectable or very low in NHEM and other melanoma cell lines. Western blot analysis shows that C8161.9 expressed the 14-3-3σ protein while it was undetectable in NHEM. We did, however, find a trace amount of protein in LOX (Figure 5). We suspect that "leaking expression" from a few spontaneously demethylated alleles within the 14-3-3σ gene may have contributed to the protein expression. This expression pattern is consistent with the 14-3-3σ gene methylation status where only C8161.9 possessed an un-methylated CpG island. The NHEM cells and remaining melanoma cell lines harbored a hypermethylated CpG island within the 14-3-3σ gene.

### Reversal of 14-3-3σ silencing following demethylation of CpG island

**Figure 6:**
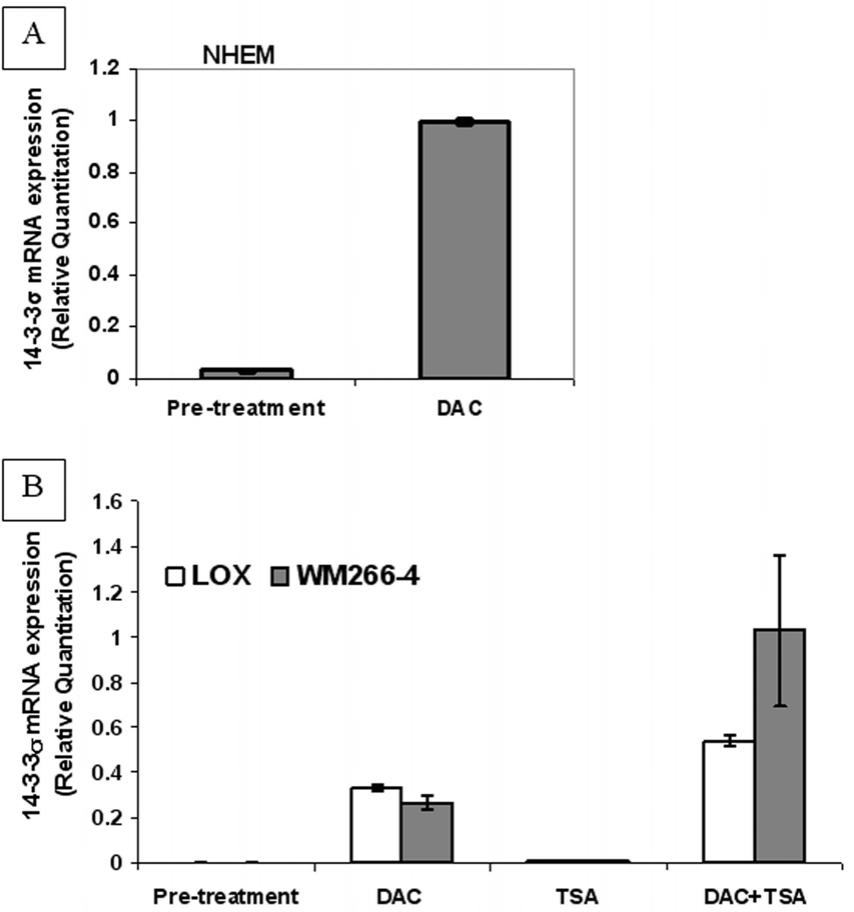
**a. Real-time PCR analysis of 14-3-3σ mRNA expression in DAC-treated NHEM**. b. Real-time PCR analysis of 14-3-3σ mRNA expression in DAC/TSA-treated melanoma cell lines LOX and WM266-4. Error bars were defined as "mean +/- SD".

**Figure 7:**
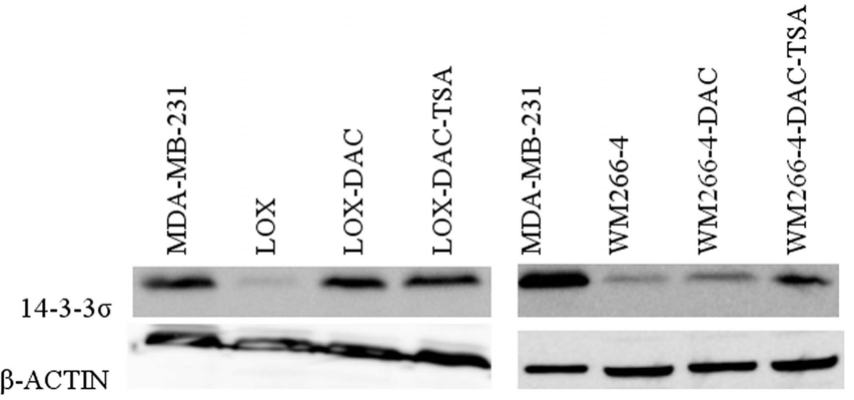
**Western blot detection of 14-3-3σ protein expression in DAC/TSA-treated melanoma cell lines LOX and WM-266-4**.

To further investigate whether CpG island methylation is the principal mechanism leading to 14-3-3σ silencing in NHEM and melanoma cell lines, we treated NHEM, LOX and WM266-4 with the demethylating agent, 5-aza-2'-deoxycytidine (DAC), alone or combined with the histone deacetylase inhibitor (HDACi), Trichostatin A (TSA). We then examined each cell line for evidence of re-expression of 14-3-3σ utilizing real-time RT-PCR and Western blot analysis. We found that after treatment with DAC, 14-3-3σ mRNA expression was significantly up-regulated in NHEM (Figure 6a) as well as LOX and WM266-4 cell lines (Figure 6b). TSA, used as a single agent, was unable to induce 14-3-3σ mRNA re-expression, but was able to exert a significant synergistic effect when combined with DAC (Figure 6b). The Western blot results are correlative with mRNA expression, as shown in Figure 7. Treatment of C8161.9 with DAC alone or combined with TSA, which possesses a fully un-methylated CpG island within the 14-3-3σ gene, was not able to further induce 14-3-3σ mRNA expressing (data not shown). Thus, our results suggest that the hypermethylation of the CpG island in the 14-3-3σ gene appears to be an active mechanism of gene silencing in both NHEM and melanoma cells.

### Hypermethylation status of the 14-3-3σ gene is maintained in melanoma samples

Our data demonstrates that the 14-3-3σ gene is hypermethylated in NHEM but hypomethylated in the highly metastatic melanoma cell line C8161.9. We therefore hypothesized that the hypermethylation status of 14-3-3σ gene may be disrupted during melanoma progression as a result of genome-wide hypomethylation, which is a known characteristic of advanced-stage tumors [14]. We next tested primary cell cultures of 10 daughter melanoma cell lines originally derived from surgically procured melanoma tissues for evidence of 14-3-3σ DNA methylation. Utilizing Q-MSP and real-time RT-PCR, we found no detectable levels of 14-3-3σ mRNA expression within any of the samples (0/10), with their CpG islands within the 14-3-3σ gene found to be highly hypermethylated. We next analyzed 20 freshly procured and cryopreserved melanoma tumor specimens from patients with stage III and IV melanoma, where melanoma cells almost completely replaced the normal tissue (see Methods). We found that all (20/20, 100%) samples exhibited significant levels of 14-3-3σ DNA hypermethylation. These results strongly demonstrate that most melanoma cells maintain intrinsic levels of hypermethylation of the 14-3-3σ gene, without concomitant levels of mRNA gene expression. Thus, the spontaneous demethylation of 14-3-3σ gene appears to be a relatively rare event in melanoma.

## Discussion

We have attempted to identify hypermethylated genes in melanoma, possibly serving as biomarkers and/or therapeutic targets for patients with advanced melanoma. The 14-3-3σ gene is notable because it has been shown to be un-methylated in normal epithelial cells but aberrantly methylated in several types of epithelial malignancies [3-9] and was indicated as a tumor suppressor gene. In contrast to other histologic cancers, here we show that the 14-3-3σ gene is hypermethylated in human melanoma in a lineage-specific manner, as it is hypermethylated in both NHEM and melanoma cells.

Several recent reports have indicated that cytosine methylation is an important event in the establishment and maintenance of cell lineage-restricted gene expression [15]. Such examples of genes that are methylated in normal cells in a lineage-specific manner are maspin [15] and MAGE genes [16]. Previously, 14-3-3σ has been reported to be hypermethylated in a cell-lineage-specific manner in non-epithelial cells such as fibroblasts, lymphocytes, chondrocytes and bone marrow [17]. Here, we report that the 14-3-3σ gene is densely methylated in human epidermal melanocytes and most melanoma cells and that CpG island hypermethylation results in the silencing of transcription of the 14-3-3σ gene in both NHEM and melanoma cells.

Transcription and expression of 14-3-3σ mRNA and protein are readily identified in the melanoma cell line C8161.9, whose CpG island in 14-3-3σ is un-methylated. At the same time, in those cell lines with a hypermethylated CpG island, DAC treatment results in the re-expression of 14-3-3σ. Significant synergistic effects are also observed when DAC is combined with TSA, with TSA treatment alone being unable to induce 14-3-3σ expression. These results strongly indicate that the 14-3-3σ gene is hypermethylated and the chromatin is not accessible due to hypoacetylated histones in NHEM and most melanoma cells.

The 14-3-3σ gene is recognized as a negative cell cycle regulator that causes G2 arrest after DNA damage [18]. Lack of 14-3-3σ gene expression may contribute to the malignant transformation via the impairment of the G2 cell cycle checkpoint function, allowing for the accumulation of gene defects within the cell. The 14-3-3σ gene has also been shown to be a regulator of mitotic translation through its direct mitosis-specific binding to a variety of translation/initiation factors [19]. The lack of 14-3-3σ gene expression leads to aberrant mitotic translation and the generation of binucleate cells. Aberrant silencing of the 14-3-3σ gene in many types of epithelial malignancies strongly suggests that it may function as a potent tumor suppressor gene. Indeed, the tumor suppression effects of 14-3-3σ in melanoma have been examined in a model cell line, MDA-MB-435. MDA-MB-435, originally thought to represent a breast cancer cell line, was later confirmed to be melanoma in origin [20]. Ferguson et al [18] demonstrated that the over-expression of 14-3-3σ led to a rapid and permanent G2 arrest in MDA-MB-435 cells. Thus re-expression of 14-3-3σ in melanoma cells may be beneficial for the control of melanoma growth, while further investigation are required to determine the functional significances of 14-3-3σ in melanoma.

## Conclusion

Here, we show that 14-3-3σ/Stratifin, as an epithelial cell antigen expressed in epithelia, is hypermethylated in both human melanocytes and most melanoma cells in a lineage-specific manner. This hypermethylation leads to silencing of 14-3-3σ expression in human melanocytes and melanoma cells. Spontaneous demethylation and re-expression of 14-3-3σ is a rare event in melanoma.

## Abbreviations

NHEM: normal human epidermal melanocytes; DAC: 5-aza-2'-deoxycytidine; TSA: Trichostatin A; MSS: Methylation-specific sequencing; Q-MSP: Quantitative methylation-specific PCR.

## Competing interests

The authors declare that they have no competing interests.

## Authors' contributions

SL conceived and designed this experiment, carried out the molecular genetic studies, drafted and modified all versions of the manuscript. SR participated in the design of the study. PH participated in the methylation-specific sequencing analysis, drafting and revisions of the final manuscript. AIR participated in the hypothesis, conception, design, oversight and coordination of all investigators and experiments, as well as drafting and revising all versions of the manuscript. All authors read and approved the final manuscript.

## Pre-publication history

The pre-publication history for this paper can be accessed here:

http://www.biomedcentral.com/1471-2407/9/162/prepub
